# Monitoring Beliefs and Physiological Measures Using Wearable Sensors and Smartphone Technology Among Students at Risk of COVID-19: Protocol for a mHealth Study

**DOI:** 10.2196/29561

**Published:** 2021-06-24

**Authors:** Christine Cislo, Caroline Clingan, Kristen Gilley, Michelle Rozwadowski, Izzy Gainsburg, Christina Bradley, Jenny Barabas, Erin Sandford, Mary Olesnavich, Jonathan Tyler, Caleb Mayer, Matthew DeMoss, Christopher Flora, Daniel B Forger, Julia Lee Cunningham, Muneesh Tewari, Sung Won Choi

**Affiliations:** 1 Division of Pediatric Hematology Oncology Department of Pediatrics University of Michigan Ann Arbor, MI United States; 2 Division of Hematology Oncology Department of Internal Medicine University of Michigan Ann Arbor, MI United States; 3 Management and Organizations Area Ross School of Business University of Michigan Ann Arbor, MI United States; 4 Department of Mathematics University of Michigan Ann Arbor, MI United States; 5 Rogel Comprehensive Cancer Center University of Michigan Ann Arbor, MI United States; 6 Department of Biomedical Engineering University of Michigan Ann Arbor, MI United States

**Keywords:** college students, COVID-19, global pandemic, mental health, mHealth, pandemic, risk monitoring, wearable sensors, well-being

## Abstract

**Background:**

The COVID-19 pandemic has significantly impacted lives and greatly affected the mental health and public safety of an already vulnerable population—college students. Social distancing and isolation measures have presented challenges to students’ mental health. mHealth apps and wearable sensors may help monitor students at risk of COVID-19 and support their mental well-being.

**Objective:**

This study aimed to monitor students at risk of COVID-19 by using a wearable sensor and a smartphone-based survey.

**Methods:**

We conducted a prospective study on undergraduate and graduate students at a public university in the Midwest United States. Students were instructed to download the Fitbit, Social Rhythms, and Roadmap 2.0 apps onto their personal smartphone devices (Android or iOS). Subjects consented to provide up to 10 saliva samples during the study period. Surveys were administered through the Roadmap 2.0 app at five timepoints: at baseline, 1 month later, 2 months later, 3 months later, and at study completion. The surveys gathered information regarding demographics, COVID-19 diagnoses and symptoms, and mental health resilience, with the aim of documenting the impact of COVID-19 on the college student population.

**Results:**

This study enrolled 2158 college students between September 2020 and January 2021. Subjects are currently being followed-up for 1 academic year. Data collection and analysis are currently underway.

**Conclusions:**

This study examined student health and well-being during the COVID-19 pandemic and assessed the feasibility of using a wearable sensor and a survey in a college student population, which may inform the role of our mHealth tools in assessing student health and well-being. Finally, using data derived from a wearable sensor, biospecimen collection, and self-reported COVID-19 diagnosis, our results may provide key data toward the development of a model for the early prediction and detection of COVID-19.

**Trial Registration:**

ClinicalTrials.gov NCT04766788; https://clinicaltrials.gov/ct2/show/NCT04766788

**International Registered Report Identifier (IRRID):**

DERR1-10.2196/29561

## Introduction

### Background

A global pandemic affecting over 100 countries has created great uncertainty, disruptions, and imposed tight restrictions on movement. This unparalleled time has required individuals to adapt to new routines, such as self-isolation, quarantine, and careful health behaviors (eg, mask-wearing, hand-washing, and self-reporting of respiratory symptoms) to protect not only one’s own health but also the health of the broader community. These routines have made it more difficult for young adults, such as college students, to see their friends and family as they were used to before the pandemic [1]. College students who are facing these unexpected changes and those who are now physically separated from friends and family are more prone to developing mental health issues in the absence of social support [1]. An additional concern is the degree to which students have had their close friends and family affected by the COVID-19 pandemic (eg, job loss, illness, and death). Accordingly, this has placed college students at a significant risk of decline in mental health and subjective well-being [1].

Having originated in Wuhan (Hubei Province, China) in the beginning of December 2019, SARS-CoV-2 was identified as the cause of an outbreak of COVID-19 [2]. As of February 2021, the virus had spread to 210 countries with more than 100 million confirmed cases [3,4]. Among those millions of cases, 76% of the confirmed cases included adults younger than 65 years, with 18-29-year-old people constituting a large proportion of the infected population [5]. This disease is usually transmitted through inhalation of or contact with infected droplets, and the incubation period ranges from 2 to 14 days [6]. Common symptoms include fever, cough, sore throat, and fatigue, among others [6]. While the illness has been mild for most people, in some—usually those with comorbidities—it progresses to pneumonia, acute respiratory distress syndrome, or multiorgan dysfunction [6]. Many people are asymptomatic but are still capable of spreading the disease, which contributes to the complexity of the disease [6,7].^ ^

During this unprecedented global pandemic, the return of students to campus has been heavily contemplated by colleges nationwide. While younger adults with COVID-19 and no other underlying health conditions are at a minor risk of severe health outcomes, colleges nationwide are making maximum efforts to halt the spread of the disease [8]. College campuses have implemented numerous COVID-19 prevention practices, including an increased testing capacity and requirements for mask-wearing and physical distancing. With these practices in place, college students are now expected to adapt to a “new normal.” This unpredictability, in conjunction with increased isolation measures, can act as stressors that may drive declines in students’ mental health [1,9].^ ^

### Rationale

In response to the COVID-19 pandemic, this study leveraged an mHealth platform in conjunction with saliva samples to monitor students at risk of COVID-19. Given the impact of the COVID-19 pandemic on college students, this study aimed to monitor student health and mental well-being. This study utilized the Roadmap 2.0 app, which is currently being evaluated among caregivers of patients undergoing hematopoietic stem cell transplantation [10-12]. The Roadmap 2.0 app was used to interface with a wearable sensor data from the Fitbit device and to deliver surveys to the subjects. This app also provided a set of positive psychology-based exercises that were available to participants to use as they desired. The study also utilized the Social Rhythms app to assess subjects’ circadian rhythms and changes that occurred as a result of the COVID-19 pandemic. 

In addition to a suite of mobile health apps, this study utilized a wearable sensor to gather physiological data continuously. Wearable devices are currently being used by millions of people worldwide and have the ability to measure physiological parameters, such as heart rate, skin temperature, and sleep in real time [13]. Given the known association between heart rate and infection, wearable sensors could be leveraged to help detect the disease and determine the health status of an individual [14,15]. Therefore, we hypothesized that continuous analysis of heart rate, sleep, and step data obtained from wearable sensors, in conjunction with COVID-19 diagnosis data and data from self-collected saliva samples, may help develop an early prediction or detection model for COVID-19 illness in students. In addition, we proposed that wearable sensor use combined with smartphone-based surveys of student health and wellness could provide a platform for students to self-monitor their well-being.

An additional challenge for monitoring students and other individuals for COVID-19 was the fact that the predominantly used specimen type for viral testing, the nasopharyngeal swab, requires sample collection by trained staff and the use of personal protective equipment because the collection process itself could make the virus airborne. Recent studies on other respiratory viruses, along with those on SARS-CoV-2, have indicated that a simple self-collected saliva sample could alternatively be used to test for infection accurately [16]. Self-collection of samples has had substantial benefits, including reducing the amount of required personal protective equipment and enabling collection at home, which could render widespread and repeated testing of students practical at the large scale. Thus, we posited that self-collected saliva samples would be feasible to collect in the college student population. This would help determine the correlation between data obtained from wearable sensors and qualitative SARS-CoV-2 positivity in saliva biospecimens. 

### Study Purpose

Our aim for this nontherapeutic pilot trial was to improve efforts of monitoring students during the COVID-19 pandemic, for whom detecting infections in the asymptomatic and presymptomatic settings could be of critical importance for the protection of the community by limiting disease spread. By using a wearable device, a smartphone-based survey, and self-collected saliva specimens, our study can potentially (1) assist students in self-monitoring for COVID-19 illness as well as mental well-being and (2) provide key data toward our ultimate goal of creating a predictive model for COVID-19 in students as well as other individuals (eg, their friends and family) in the future.

## Methods

### Study Design

#### Overview

**Figure 1:**
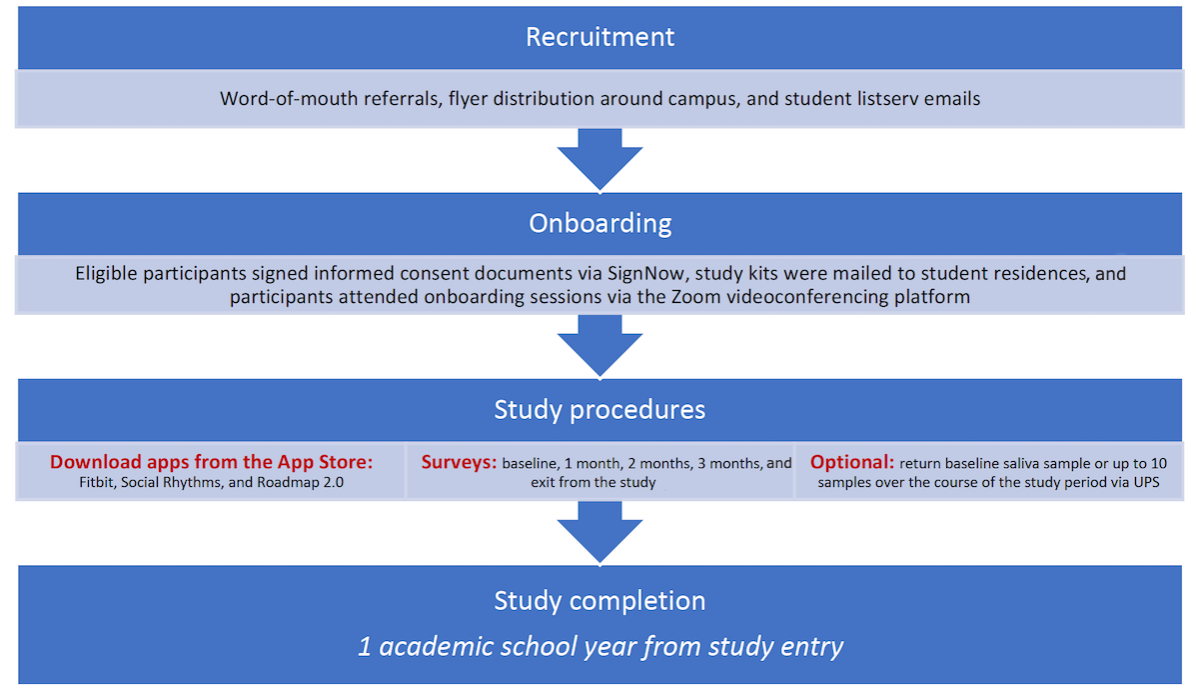
Schematic representation of the study protocol. UPS: United Parcel Service.

This was a prospective study on students at the University of Michigan (Ann Arbor, Michigan). We aimed to enroll up to 5000 subjects in this study. Subjects self-collected a saliva sample at baseline and were asked to provide up to 10 samples throughout the academic year by using an easy-to-use at-home collection kit. In addition to samples, subjects were instructed to download the Fitbit, Social Rhythms, and Roadmap 2.0 apps on their smartphone devices (Android and iOS). Surveys were administered through the Roadmap 2.0 app at baseline, on a monthly basis for 3 months, and at study completion, and participants received push notifications as a reminder to complete the survey through the app. Subjects were unable to review or change any of their answers while taking their surveys. The study schema is shown in Figure 1.

#### Objectives

The primary objective of this study was to assess the feasibility of using wearable devices among college students. Feasibility was defined as wearing the Fitbit Charge 3 watch at least 8 hours per day up to at least 5 days of the week (~40 hours/week).

The secondary objective was to assess the survey completion rate, assuming that at least 50% of participants would complete the baseline, monthly, and exit surveys.

The exploratory objectives were to (1) analyze the time spent in performing the positive activities and data on circadian rhythms (by analyzing time stamps) and correlate mental health status to positive activity usage, (2) analyze continuous heart rate data from a wearable device alongside intermittent saliva samples for the development of an early prediction and detection model for COVID-19, and (3) identify individuals who may have already been exposed to COVID-19, once validated serologic tests for COVID-19 exposure are available for research purposes only.

### Participant Enrollment 

#### Eligibility Criteria

Subjects were required to be at least 18 years of age or older and attend the University of Michigan. Undergraduate or graduate students could either be on campus for the academic year or taking classes on the internet. Students completing school remotely were required to provide a mailing address within the United States where they could receive study material (eg, Fitbit and saliva collection kits). In addition, subjects were required to possess a smartphone device (Android or iOS). Finally, subjects were required to understand and demonstrate a willingness to sign a written informed consent document remotely. Subjects were excluded from the study if they were unwilling or unable to comply with the study procedures.

#### Recruitment

The primary tools for recruitment were word-of-mouth referrals, flyer distribution around campus, and student listserv emails. We listed this study on the University of Michigan Health Research recruitment website, and we obtained permission from the School of Public Health to advertise on their job board and student flyers. Interested participants notified a study email address, or they used the QR code on the recruitment flyer to indicate their interest. In addition, the study team requested a targeted email to be sent out to all University of Michigan students, which captured other student populations on or off campus, and approval was obtained from the Registrar’s Office. 

#### Informed Consent

Following the social distancing guidelines issued by the Centers for Disease Control and Prevention, as well as the University of Michigan COVID-19 research guidelines, we conducted an entirely remote process for obtaining informed consent. Interested subjects who contacted the study team by phone or email received additional study information (ie, overview of study procedures, risks, benefits, etc). Following adequate discussion with the coordinator to answer any questions, the coordinator sent the informed consent documents via email through the SignNow platform, and the student signed the document electronically [17].

#### Enrollment 

After signing the informed consent document through SignNow, the study coordinators assembled the study kit, which included the wearable Fitbit device and a saliva collection kit. Each study kit contained written directions for collecting the saliva sample and biohazard packaging to safely return the sample to the laboratory via the United Parcel Service. Once the subject received his/her study kit, the coordinators sent an email that included the dates and times of live onboarding sessions conducted via the Zoom videoconferencing, as well as a link to a prerecorded web-based onboarding video if they could not attend the live onboarding session. During web-based onboarding, a study coordinator instructed subjects on how to download the Fitbit, Roadmap 2.0, and Social Rhythms apps. The study coordinator then explained each app and explained how to use and sync their Fitbit watch. The baseline survey was also completed through the Roadmap 2.0 app. Finally, instructions on collecting and mailing the saliva sample were provided to subjects. 

### Biospecimens

#### Saliva Sample Collection

Subjects were asked to collect a saliva samples at baseline and were asked to collect up to 10 samples throughout the study. When signing the consent documents, subjects could choose to opt in or out of saliva sample collection. Subjects were provided the Zymo DNA/RNA Shield™ Saliva Collection Kit to self-collect their samples at home [18]. When providing a sample, subjects collected their saliva specimens prior to brushing their teeth, eating, or drinking, and collected approximately 1-2 mL of saliva in a tube that contained no preservative. They then added a preservative (DNA/RNA) shield reagent, which effectively preserved the nucleic acid content of the sample [19].^ ^

#### Specimen Handling, Processing, and Storage

Owing to the unknown COVID-19 status of subjects while they were enrolled and provided biospecimens for this study, our team developed a sample handling procedure to ensure the safety of all laboratory members. All activities in the laboratory were performed with disposable gloves while wearing safety glasses, a coat, and a face mask. All samples arriving at the laboratory were placed in the biosafety cabinet. Once in the biosafety cabinet, both the external packaging and primary sample container were sprayed with 70% ethanol and left to stand for 5 minutes before being stored at –80°C. When finished working in the biosafety cabinet, the cabinet was sprayed down with 70% ethanol, and the UV light in the biosafety cabinet was turned on for 15 minutes.

### Wearable Devices

Fitbit Charge 3 is an advanced touch-screen fitness tracker that monitors variables such as heart rate, calories burned, pace, distance, and sleep cycles. The device can be worn either on the wrist and wirelessly connects with the patient’s smartphone via Bluetooth to the Fitbit mobile app (available on Android and iOS systems). Subjects had access to their data and, if they wanted, could set fitness goals within the app.

The subject was instructed to wear the Fitbit device for at least 8 hours per day up to at least 5 days of the week. The smartwatch uploaded data to the subject’s phone every 15 minutes via Bluetooth Low Energy [20]. The study team had access to the patient dashboard and data download for analysis through the Roadmap App. In addition to the study team, subjects also had access to view their health data in real time on their Fitbit app.

### Apps Used in This Study

#### Roadmap 2.0 App

Each subject enrolled in the study was asked to download the Roadmap 2.0 app on their smartphone. The Roadmap 2.0 app was developed by University of Michigan investigator SWC and colleagues, and it served as an interface with the Fitbit app and provided activities designed to promote subjective well-being, based on the principles of positive psychology [21]. The activities were as follows: positive piggy bank, gratitude diary, savoring, pleasant activity, random acts of kindness, signature strengths, love letters, and engaging with beauty (Multimedia Appendix 1). On the app, participants were provided with graphs that showcase their daily mood, sleep, and steps data. The quality and quantity of the graphs data depended on whether the participants responded to the daily mood questionnaire—a scale with scores ranging between 1 and 10, corresponding to responses of “worst possible” and “best possible,” respectively—and wore and synced their Fitbit device. 

#### Social Rhythms App

Each subject was instructed to download the Social Rhythms app. The Social Rhythms app was developed by University of Michigan investigator DBF and colleagues, and it provides information regarding one’s circadian rhythm based on physiological data, which could inform how to organize daily schedules. Social rhythms are patterns of daily behaviors that could perhaps affect circadian timing directly or indirectly by modifying exposure to light [22]. The app collected data from either the Apple Health Kit on the app or Fitbit (health, sleep, and activity data). The app used these data to generate a report to the user about their circadian rhythm and supplied the report after a few days [23]. The report provided the user information on his/her activity pattern, regularity, internal clock, sleep time, light exposure, and documents on when the user should avoid light [23]. The user can submit his/her data and obtain a report at his/her preferred frequency. 

### Surveys

#### Baseline Surveys 

During study onboarding, subjects were provided with a unique access code that allowed them to log in to the Roadmap 2.0 app. After entering the access code, the baseline survey was automatically pushed to their smartphones, and survey completion was required in order to gain access to the rest of the app. The baseline survey consisted of 192 survey items that gathered demographic data (Multimedia Appendix 2) and were distributed on 12 pages. The estimated time of completion was less than 25 minutes. This was a Qualtrics-based survey that was stored on HIPAA-compliant University of Michigan secure servers. Study subjects also completed a 9-item baseline survey in the Social Rhythms app, which gathered demographic data through their smartphone, which took less than 5 minutes to complete. 

#### Monthly Survey

Study subjects completed a 127-item monthly survey at 1 month, 2 months, and 3 months after study entry, which were distributed on 12 pages. The survey gathered information about COVID-19 symptom tracking and mental well-being and resilience. Monthly surveys took approximately 15-20 minutes to complete. When subjects completed the study, there was also a final exit survey that had up to 60 items, which were distributed on 5 pages, regarding the number of COVID-19 tests received, COVID-19–related symptoms, vaccination status, and well-being (eg, COVID-19 compassion, fatigue, and hope), which was expected to take approximately 5 minutes to complete. Certain survey items were only conditionally displayed on the basis of responses to other items, including the number of COVID-19 tests conducted. Subjects were not able to review or change their survey responses. Subjects were compensated US $10 for completing the baseline survey and each additional monthly survey, with a total compensation of up to US $40. They were also allowed to keep the Fitbit device for personal use after study completion.

### Data Collection and Analysis

#### Quality Assurance 

The study team monitored device data regularly to assess data quality and subject compliance. When a subject consented to the device portion of the study, the coordinator discussed the subject’s preferred methods of contact in the event of poor data quality, lack of data over an extended period of time, or other technical issues. The study team sent out weekly follow-up reminders via email to subjects to complete their surveys and return saliva samples. 

#### Data Analysis Plan

Descriptive statistics will be used to analyze the subjects’ characteristics including age, gender, race, ethnicity, education (years), and comorbidities. We aim to use computational techniques to assess the relationship among self-reported symptom data, continuous heart rate data, and the incidence of clinical respiratory illness, which may be diagnosed as COVID-19 or other types of infections. We will build on analytic approaches already developed in our study on oncology patients (ie, patients undergoing hematopoietic stem cell transplantation, who develop fevers leading to changes in heart rate, step, and sleep owing to infections, graft-versus-host disease, or other causes; patients receiving chimeric antigen receptor T cells, who develop cytokine release syndrome, display some similarity to the pathophysiology of severe COVID-19) [24,25]. 

In brief, we shall adopt a multitiered approach to data analysis. This will involve initial quality control analysis and nonspecific filtering of the data, data visualization, descriptive statistics, and multivariate analysis to calculate measures of correlation among the multiple streams of data themselves (eg, heart rate and steps) as well as with clinical outcomes (eg, COVID-19 status). We will consider examining such relationships, especially clinical outcomes, as exploratory and expect to obtain pilot data to power a subsequent study. We also aim to assess the relationship between student mental well-being and the use of the study apps, including Roadmap 2.0, Fitbit, and Social Rhythms. 

## Results

This study was approved by the institutional review board on August 20, 2020, at Michigan Medicine (Ann Arbor, Michigan) and was registered on ClinicalTrials.gov (NCT04766788). We enrolled a total of 2158 students in this study between September 24 and November 10, 2020. This sample included both undergraduates and graduate students. The entire study was conducted remotely owing to COVID-19 restrictions. Survey data collection remains active until the last subject completes the study procedures by June 2021. As of this writing, subjects will remain enrolled in this study for 1 academic year (August 2020 to May 2021: fall term 2020 and winter term 2021), during which they will continue wearing a noninvasive Fitbit device. Hence, data collection, processing, and analyses are ongoing. 

## Discussion

In this study, college students were instructed on the use of a smartwatch, downloaded several mobile apps, submitted saliva samples, and completed smartphone-based surveys with the aim of helping them self-monitor for COVID-19 infection and overall mental well-being. We enrolled 2158 students, of whom 1609 have completed the final exit survey to date. This study assessed the feasibility of wearable sensor use and survey completion among college students. This study also examined the effects of study app usage on mental well-being. Thus far, we have been encouraged by the high interest and engagement of college students who are willing to participate in the study, as well as the number of students who have followed through with each of the study procedures.

This long-lasting pandemic has affected college students worldwide in numerous ways. The pandemic has brought about major lifestyle changes and constraints on socialization for students. These stressors have highlighted the precarious mental health of this vulnerable population [26]. Recent studies have shown that longer periods of quarantine were linked to a decline in mental health [27]. Some other stressors have included fears of infection, frustration, boredom, and financial losses [28]. Given the critical importance of student mental well-being, beliefs, and resilience during this unprecedented time, this study aimed to provide them with tools that could enhance mental well-being.

With the growing use of smartphones and mobile apps, these tools are increasingly being used to target healthy behaviors and manage health conditions [26,29]. Previous studies have elucidated the important role of mobile apps in the maintenance of mental health [30,31]. Considering this, we designed this study to leverage an mHealth platform (Roadmap 2.0 app + Fitbit devices + symptom reporting) to monitor COVID-19 symptoms and student mental well-being. As the literature on mHealth interventions continues to grow, wearable sensors and mobile health technologies are likely to play an increasingly important role in the lives of college students. A recent study on college students tested a smartphone app, Nod, which was designed to deliver cognitive and behavioral skill-building exercises to reduce loneliness during the transition to college. The findings of their pilot randomized controlled trial suggest the unique potential of Nod to provide self-paced and confidential support in addressing mental health issues including depression and loneliness [32]. Indeed, by examining real-time data, mobile health apps and wearable sensors have the potential to inform real-time decision-making. Furthermore, these new tools and technologies may help improve access to care, care quality, patient-provider communication, and health outcomes [33]. Importantly, mHealth tools may serve as a cost-effective and scalable solution for deploying novel interventions that can impact the lives of people with physical and mental health conditions [34].

We acknowledge various limitations of this study. First, this study was limited to a single college campus setting. Making this study accessible to other campuses would help capture the diversity of other student bodies in the United States. Second, studies have shown that the usage of mHealth apps is often short-lived; one study found that among people who have downloaded an mHealth app, approximately half of that population deleted the app for various reasons including hidden costs, loss of interest, and high data entry burden [35]. Thus, future studies are required to address these barriers to mHealth app usage to increase engagement and sustainment of use. Finally, college students may experience fatigue and lose motivation when completing monthly surveys, which can take up to 30 minutes to complete. Thus, to keep subjects engaged, surveys could perhaps be shortened or be conducted less frequently. Despite the aforementioned limitations, this study will provide key pilot data toward developing novel tools, technologies, and methodologies for supporting student mental well-being, particularly during stressful life events, such as a global pandemic, examinations, presentations, or job interviews. 

## Appendix

Multimedia Appendix 1 Screenshots of Roadmap 2.0.

Multimedia Appendix 2 Survey Measures.
