# Supplementary material for: Monitoring Beliefs and Physiological Measures Using Wearable Sensors and Smartphone Technology Among Students at Risk of COVID-19: Protocol for a mHealth Study
Source: JMIR Res Protoc. 2021 Jun 24;10(6):e29561. doi: 10.2196/29561 (PMC8386373; doi:10.2196/29561)
Supplement: Multimedia Appendix 1 [file resprot_v10i6e29561_app1.docx]

**Multimedia Appendix I. Screenshots of Roadmap 2.0**


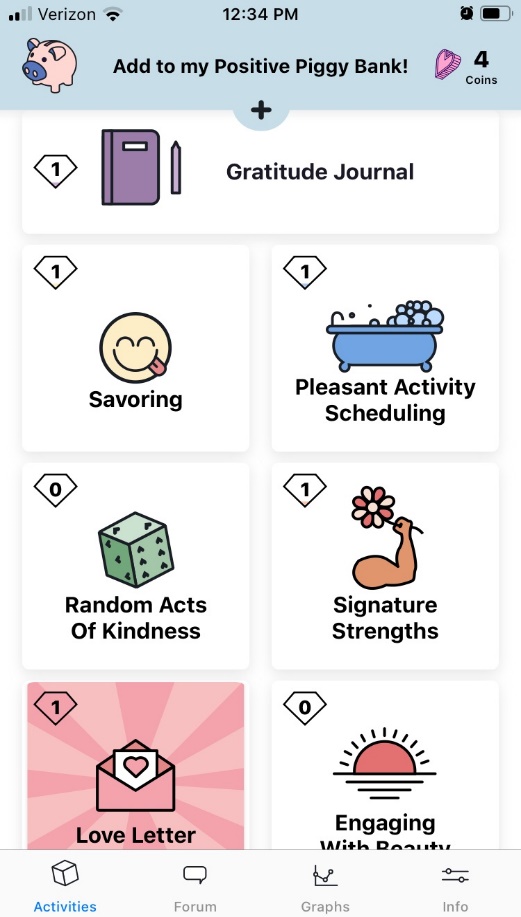

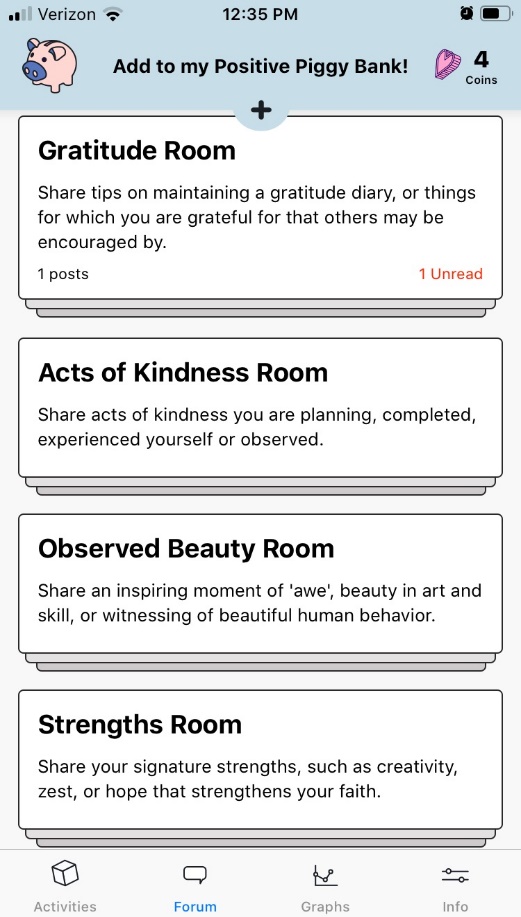

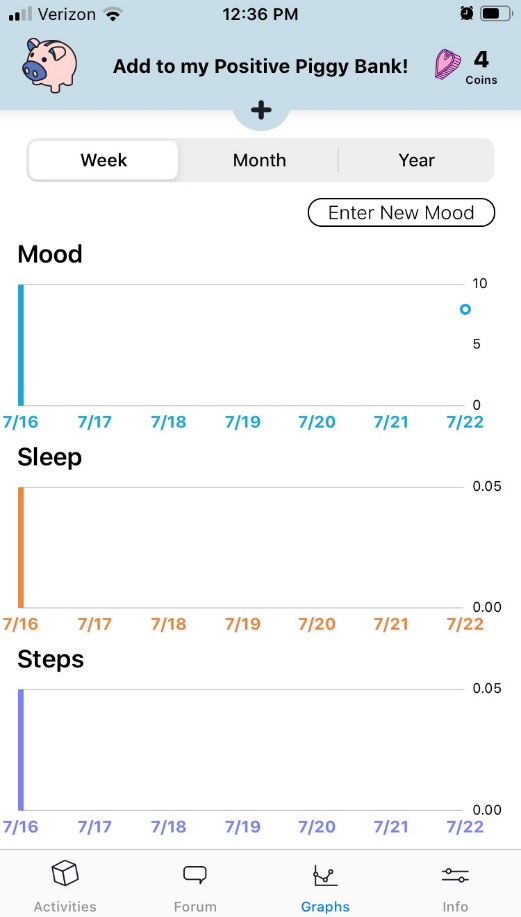


**FITNESS TRACKER + DAILY MOOD**

**CHAT**

**FORUM**

**POSITIVE**

**ACTIVITIES**

**Gratitude Journal**

**
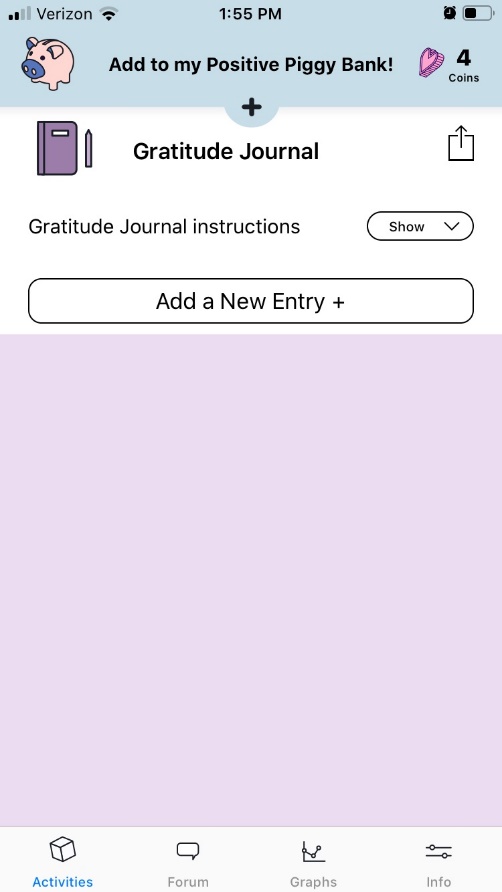

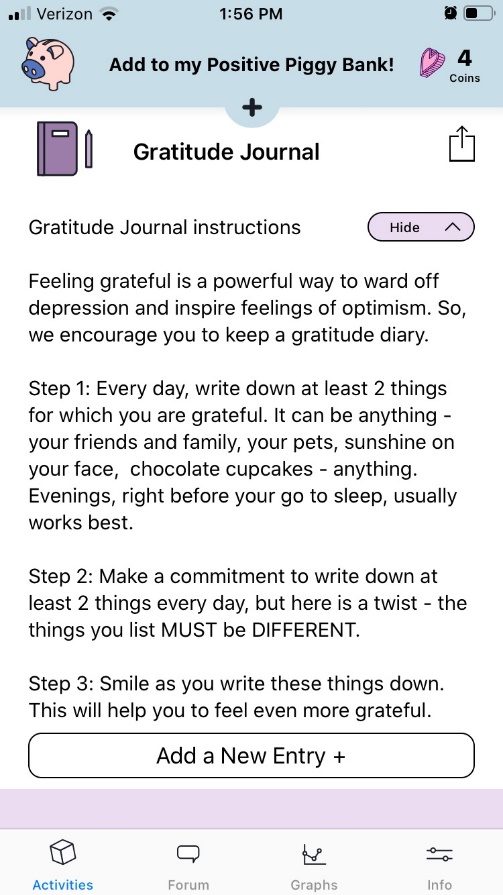

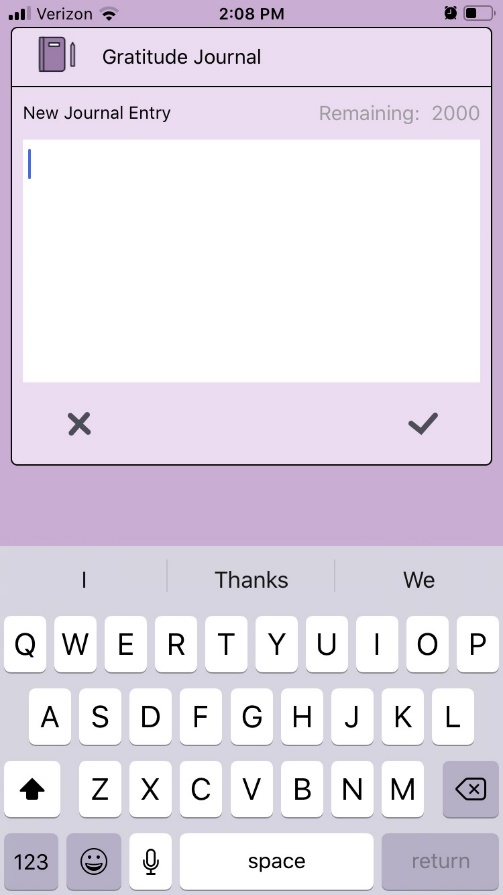
**

**Engaging with Beauty**

**
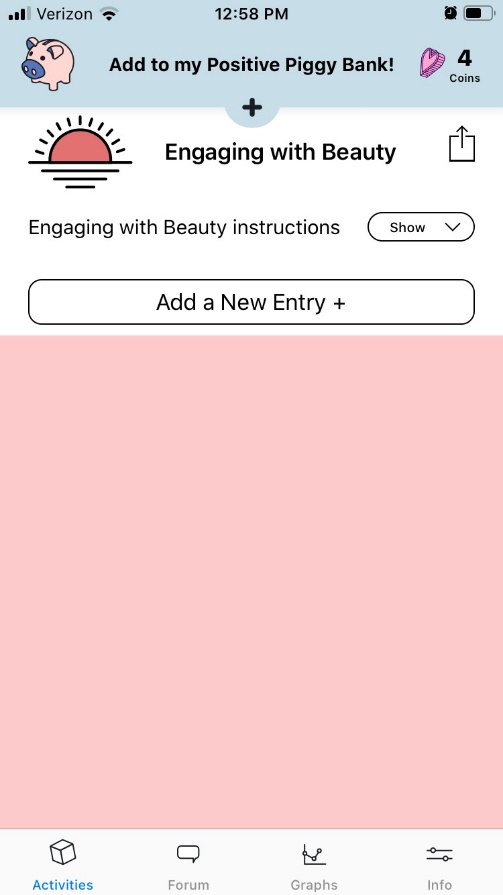

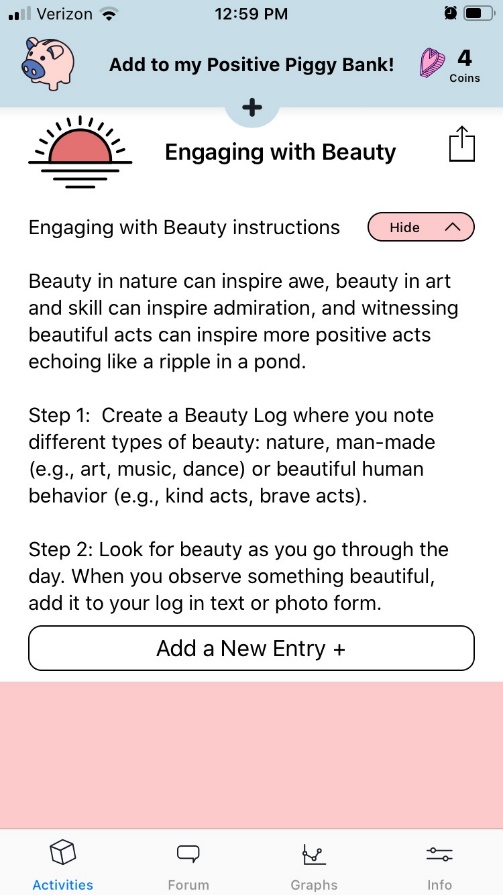

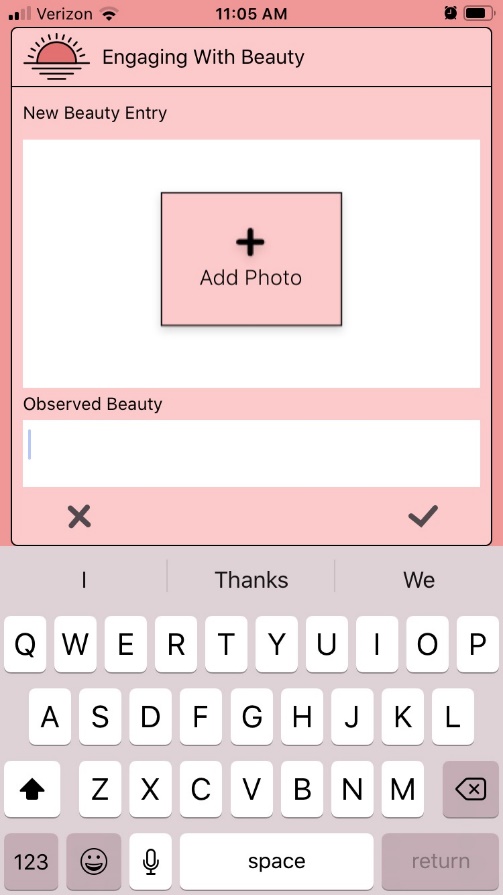
**

**Savoring**

**
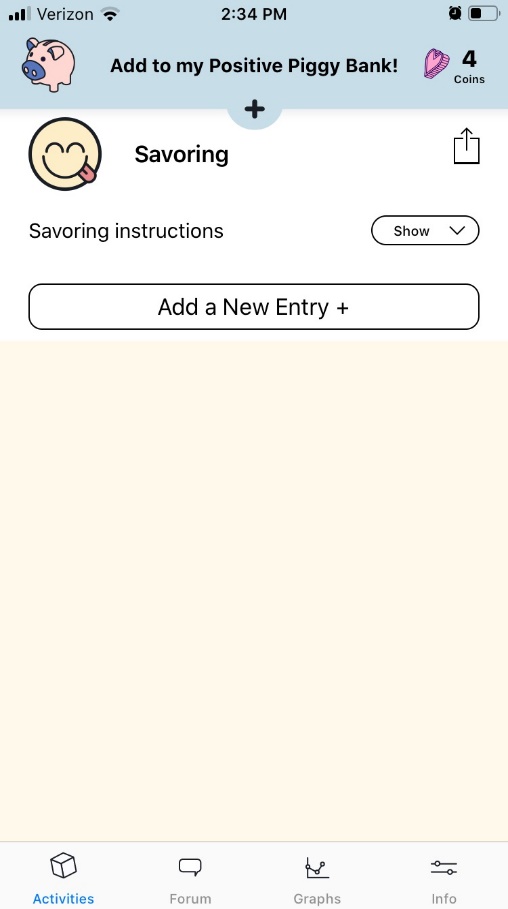

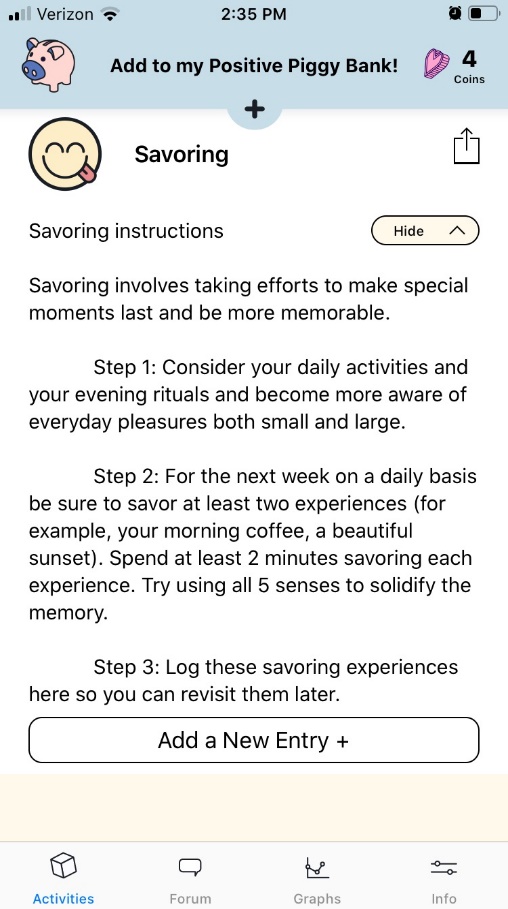

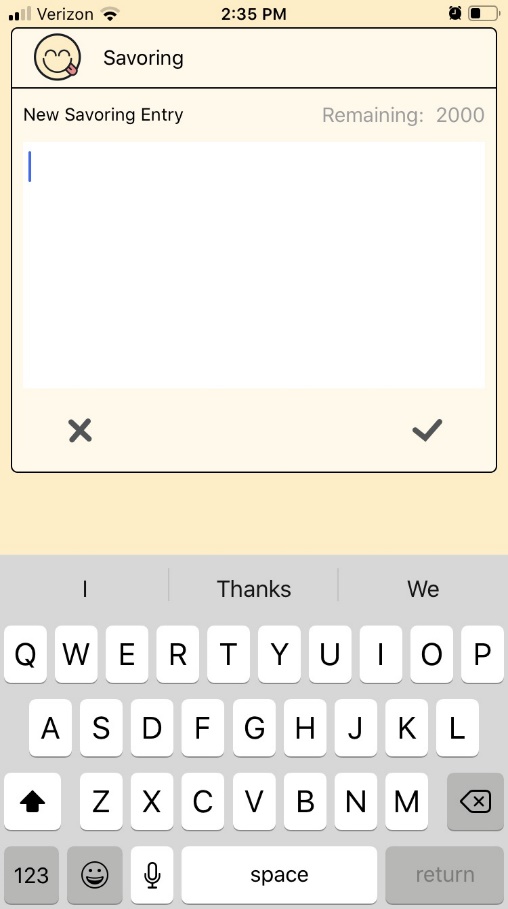
**

**Signature Strengths**

**
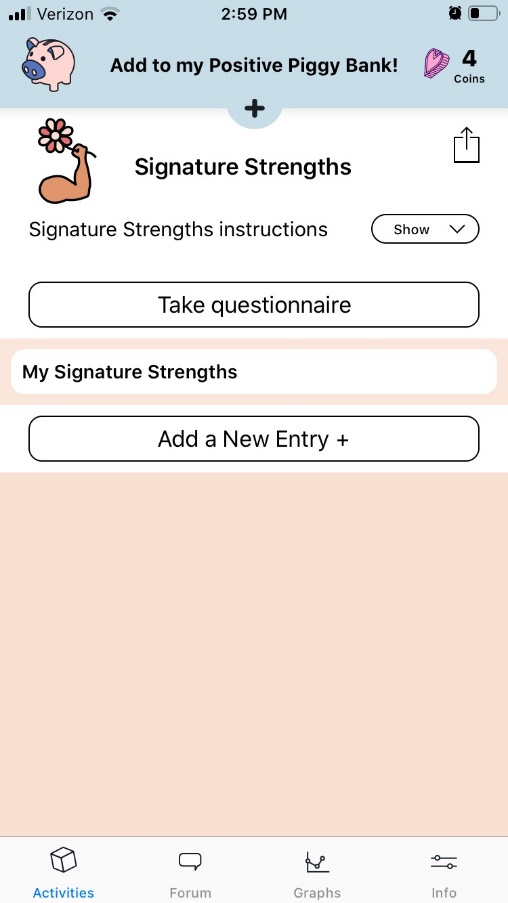

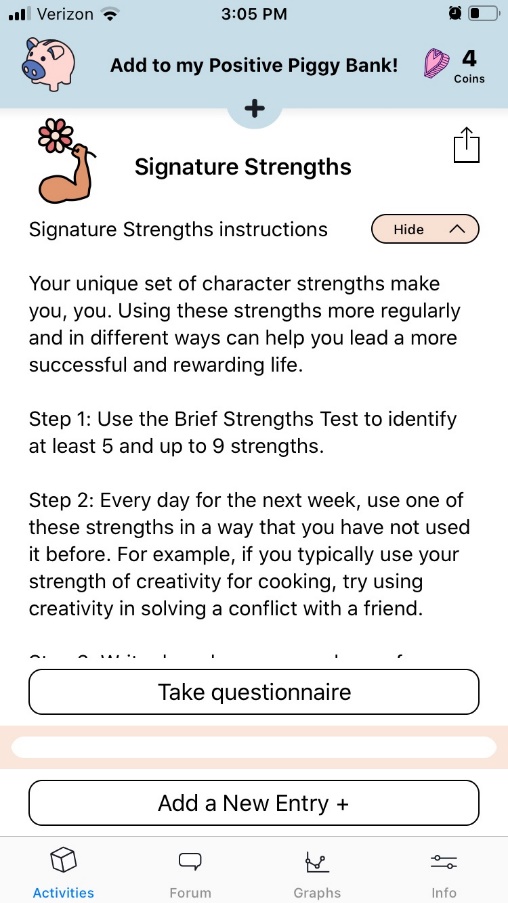
**
