# Supplementary material for: Monitoring Beliefs and Physiological Measures Using Wearable Sensors and Smartphone Technology Among Students at Risk of COVID-19: Protocol for a mHealth Study
Source: JMIR Res Protoc. 2021 Jun 24;10(6):e29561. doi: 10.2196/29561 (PMC8386373; doi:10.2196/29561)
Supplement: Multimedia Appendix 2 [file resprot_v10i6e29561_app2.docx]

**Multimedia Appendix II: Survey Measures**

Baseline Survey

1. What year of schooling is this?

a. Freshman

b. Sophomore

c. Junior

d. Senior

e. First-year graduate student

f. Second-year or above graduate student

g. Other; please specify ____________

2. What is your gender?

a. Female

b. Male

c. Not Listed (Please specify ___________)

3. Are you of Spanish, Hispanic, or Latino origin or descent?

a. Yes

b. No

4. What is your race? (choose all that apply)

a. White

b. Black or African American

c. Asian

d. Native Hawaiian or other Pacific Islander

e. American Indian or Alaska Native

f. Other (please specify) __________

5. Are you a domestic or international student?

a. Domestic

b. International

6. Are you a first generation student or continuing generation student?

a. First generation

b. Continuing generation

7. How are you financing your education? Please select all that apply.

a. Government loans

b. Federal Grants

c. Merit-based scholarship

d. Parents or family financed

e. Self-financed

f. Other; please specify

8. What is your current housing?

a. Living at home with parents or family members

b. Living on/near campus in a house

c. Living on/near campus in an apartment

d. Living on campus in a dorm

e. Living further than 1 hour drive away from Ann Arbor, but not at family’s home

f. Other; please specify _______________

9. What is the makeup of the people living in your household (check all that apply and include number)

a. Roommates (Same room):___

b. Roommates/housemates (different room): ___

c. Family: ___

d. I live alone

10. Have you been diagnosed with COVID19?

a. Yes

b. No

11. If yes: What was the approximate of diagnosis? ___________

12. If yes: Did you require hospitalization for COVID19?

a. Yes

b. No

13. If you required hospitalization, how long were you hospitalized? (Please indicate if hospitalization occurred more than once) _______________

14. Please select the symptoms that you experienced while having COVID19.

a. Fever

b. Chills

c. Shortness of breath

d. New or worsening cough

e. Sore throat

f. Body aches

g. Vomiting

h. Diarrhea

i. Loss of smell

j. Loss of taste

k. None of the above

l. Other

15. How would you rate your overall health during the past year?

a. Very Poor Excellent

1 2 3 4 5 6 7

16. How would you rate your overall quality of life during the past year?

Very Poor Excellent

1 2 3 4 5 6 7

17. Do you have any of the following health conditions? (please check all that apply)

a. Asthma

b. COPD

c. Obstructive sleep apnea

d. Pulmonary hypertension

e. Other lung disease (please specify): _________

f. Diabetes

g. Heart disease

h. High blood pressure

i. High cholesterol

j. Kidney disease

k. Cancer

l. Hepatitis

m. Fatty liver disease

n. Crohn’s disease or ulcerative colitis

o. Multiple sclerosis (MS)

p. Lupus or other autoimmune condition

q. Stroke

r. Arthritis

s. Migraine headaches

t. Seasonal allergies

u. Insomnia or other sleep disorder

v. Anxiety

w. Depression

x. Post-traumatic stress disorder

y. Other mental health disorder

z. Brain injury

aa. Other (please specify) __________

bb. None of the above

18. Do you live with someone who falls into the high-risk category for COVID19 (e.g., elderly, immunocompromised, multiple health problems)?

a. Yes

b. No

c. Unsure

19. Do you currently smoke cigarettes?

a. Yes

b. No

20. If yes, number of years smoking cigarettes: ______

21. If yes, average number of pack(s) smoked per day:__________Pack(s) of cigarettes

Within the past year how often have you….

22. Used Marijuana

a. Did not use

b. Once/Year

c. 6 times/Year

d. Once/Month

e. Twice/Month

f. Once/Week

g. Three times/Week

h. Every Day

23. Engaged in vaping (Juul, e-cigarettes, weed pens, etc.)

a. Did not use

b. Once/Year

c. 6 times/Year

d. Once/Month

e. Twice/Month

f. Once/Week

g. Three times/Week

h. Every Day

24. Consumed alcohol

a. Did not use

b. Once/Year

c. 6 times/Year

d. Once/Month

e. Twice/Month

f. Once/Week

g. Three times/Week

h. Every Day

25. In the last 12 months how often have you participated in some form of active physical exercise (e.g., running, yoga, pilates, cross-fit, dance)?

a. Daily

b. 3 to 4 times per week

c. 1 to 2 times per week

d. 1 to 2 times per month

e. Not at all

Questions related to the experience and value from participating in the study; questions related to beliefs and expectations.

26. I feel stressed about the COVID-19 pandemic.

a. Strongly agree (1)

b. Somewhat agree (2)

c. Neither agree nor disagree (3)

d. Somewhat disagree (4)

e. Strongly disagree (5)

27. I feel optimistic about the future

a. Strongly agree (1)

b. Somewhat agree (2)

c. Neither agree nor disagree (3)

d. Somewhat disagree (4)

e. Strongly disagree (5)

28. I feel I am likely to become infected with the novel coronavirus.

a. Strongly agree (1)

b. Somewhat agree (2)

c. Neither agree nor disagree (3)

d. Somewhat disagree (4)

e. Strongly disagree (5)

f. I have already had a positive COVID-19 test

29. I feel safe from COVID-19.

a. Strongly agree (1)

b. Somewhat agree (2)

c. Neither agree nor disagree (3)

d. Somewhat disagree (4)

e. Strongly disagree (5)

30. I believe I will remain healthy with respect to COVID-19

a. Strongly agree (1)

b. Somewhat agree (2)

c. Neither agree nor disagree (3)

d. Somewhat disagree (4)

e. Strongly disagree (5)

31. Approximately how many times per day have you checked the news on average during the last 2 weeks?

a. 0 -1 (1)

b. 2 - 4 (2)

c. 5 - 7 (3)

d. > 7 (4)

32. What is your primary type of cell phone?

a. Apple iPhone

b. Android smartphone (e.g., Samsung, Google)

c. Microsoft windows smartphone (e.g., Microsoft, HP Elite)

d. Cell phone that is not a smartphone

e. Other (please specify) __________

33. Do you own a fitness or smart watch? (check all that apply)

a. Apple iWatch

b. Fitbit Fitness Watch

c. Garmin Fitness Watch

d. Other (please specify) _________

e. I do not own a fitness or smart watch

34. Which of the following kinds of health or wellness-related apps do you use on your smartphone or tablet? (please check all that apply)

a. Fitness workouts

b. Counting steps

c. Nutrition (e.g., tracking calories, recording diet)

d. Meditation or stress management

e. Sleep

f. Other (please specify) __________

35. Over the last 2 weeks, how often have you been bothered by feeling nervous, anxious or on edge?

a. Not at all

b. Several days

c. More than half the days

d. Nearly everyday

36. Over the last 2 weeks, how often have you been bothered by not being able to stop or control worrying?

a. Not at all

b. Several days

c. More than half the days

d. Nearly everyday

37. Over the last 2 weeks, how often have you been bothered by little interest or pleasure in doing things?

a. Not at all

b. Several days

c More than half the days

d. Nearly everyday

38. Over the last 2 weeks, how often have you been bothered by feeling down, depressed, or hopeless?

a. Not at all

b. Several days

c. More than half the days

d. Nearly everyday

While these surveys will not be processed immediately. If a participant, indicates d to question #38 “Over the last 2 weeks, how often have you been bothered by feeling down, depressed, or hopeless?” the following message will be provided at the end of the survey;

“U-M Counseling & Psychological Services (CAPS), offers free, confidential services for U-M students including: short term counseling for individual students or couples. For urgent concerns, walk-in and 24-hour phone support (call 734-764-8312).

COVID related questions

34. In the last month or since you took the last survey, have you been tested for COVID19 (diagnostic and/or antibody test)?

a. Yes

b. No

35. What type of testing have you done? (if yes to being tested)

a. Swab for PCR detection

b. Serology/ antibody

c. Other

36. What was the result of the serology testing? (if yes to serology)

a. Positive

b. Negative

c. Test results are still Pending

37. What was the result of the PCR testing? (if yes to PCR testing)

a. Positive

b. Negative

c. Test results are still Pending

38. If Yes to Question 36 or 37 (i.e., positive COVID-19 test results): What was the approximate date of diagnosis? __________

39. In the last month or since you took the last survey, have you been around someone who has been or is presumed to have COVID19?

a. Yes

b. No

Psychological Questionnaires

BRIEF COPE (Given at both baseline and monthly surveys)

These items deal with ways you've been coping with the stress in your life since you found out you were going to have to have this operation. There are many ways to try to deal with problems. These items ask what you've been doing to cope with this one. Obviously, different people deal with things in different ways, but I'm interested in how you've tried to deal with it. Each item says something about a particular way of coping. I want to know to what extent you've been doing what the item says. How much or how frequently. Don't answer on the basis of whether it seems to be working or not—just whether or not you're doing it. Use these response choices. Try to rate each item separately in your mind from the others. Make your answers as true FOR YOU as you can.

a = I haven't been doing this at all

b = I've been doing this a little bit

c = I've been doing this a medium amount

d = I've been doing this a lot

I've been turning to work or other activities to take my mind off things.

2. I've been concentrating my efforts on doing something about the situation I'm in.

3. I've been saying to myself "this isn't real.".

4. I've been using alcohol or other drugs to make myself feel better.

5. I've been getting emotional support from others.

6. I've been giving up trying to deal with it.

7. I've been taking action to try to make the situation better.

8. I've been refusing to believe that it has happened.

9. I've been saying things to let my unpleasant feelings escape.

10. I’ve been getting help and advice from other people.

11. I've been using alcohol or other drugs to help me get through it [COVID-19 pandemic].

12. I've been trying to see it in a different light, to make it seem more positive.

13. I’ve been criticizing myself.

14. I've been trying to come up with a strategy about what to do.

15. I've been getting comfort and understanding from someone.

16. I've been giving up the attempt to cope.

17. I've been looking for something good in what is happening.

18. I've been making jokes about it.

19. I've been doing something to think about it less, such as going to movies, watching TV, reading, daydreaming, sleeping, or shopping.

20. I've been accepting the reality of the fact that it has happened.

21. I've been expressing my negative feelings.

22. I've been trying to find comfort in my religion or spiritual beliefs.

23. I’ve been trying to get advice or help from other people about what to do.

24. I've been learning to live with it.

25. I've been thinking hard about what steps to take.

I’ve been blaming myself for things that happened.

27. I've been praying or meditating.

28. I've been making fun of the situation

While these surveys will not be processed immediately. If a participant, indicates b, c, or d to question #16 “I’ve been giving up the attempt to cope” the following message will be provided at the end of the survey;

“U-M Counseling & Psychological Services (CAPS), offers free, confidential services for U-M students including: short term counseling for individual students or couples. For urgent concerns, walk-in and 24-hour phone support (call 734-764-8312)

Loneliness Scale (Given at baseline and monthly)

1. How often do you feel that you lack companionship? (hardly ever, some of the time, often)

2. How often do you feel left out? (hardly ever, some of the time, often)

3. How often do you feel isolated from others? (hardly ever, some of the time, often)

Flourishing Scale (Given at baseline and monthly)

The “Flourish” measure is obtained by summing the scores from each of the first five domains. The “Secure Flourish” measure is obtained by summing the scores from all six domains including the financial and material stability domain. Each of the questions is assessed on a scale of 0–10.

Domain 1: Happiness and Life Satisfaction.

Overall, how satisfied are you with life as a whole these days?

• 0 = Not Satisfied at All, 10 = Completely Satisfied

In general, how happy or unhappy do you usually feel?

• 0 = Extremely Unhappy, 10 = Extremely Happy

Domain 2: Mental and Physical Health.

In general, how would you rate your physical health?

• 0 = Poor, 10 = Excellent

How would you rate your overall mental health?

• 0 = Poor, 10 = Excellent

Domain 3: Meaning and Purpose.

Overall, to what extent do you feel the things you do in your life are worthwhile?

• 0 = Not at All Worthwhile, 10 = Completely Worthwhile

I understand my purpose in life.

• 0 = Strongly Disagree, 10 = Strongly Agree

Domain 4: Character and Virtue.

I always act to promote good in all circumstances, even in difficult and challenging situations.

• 0 = Not True of Me, 10 = Completely True of Me

I am always able to give up some happiness now for greater happiness later.

• 0 = Not True of Me, 10 = Completely True of Me

Domain 5: Close Social Relationships.

I am content with my friendships and relationships.

• 0 = Strongly Disagree, 10 = Strongly Agree

My relationships are as satisfying as I would want them to be.

• 0 = Strongly Disagree, 10 = Strongly Agree

Domain 6: Financial and Material Stability.

How often do you worry about being able to meet normal monthly living expenses?

• 0 = Worry All of the Time, 10 = Do Not Ever Worry

How often do you worry about safety, food, or housing?

• 0 = Worry All of the Time, 10 = Do Not Ever Worry

Social Identity Affirming Behaviors (1=not at all, 5= always)- Given at baseline and monthly

· I find creative new ways to maintain my old routines (e.g., video chats with family and friends; online exercise classes; cultural activities online).

• I engage with “virtual communities” through social media and online groups to replace the in-person communities I can no longer be a part of.

Beliefs about compassion scale 1 & 2 (1=not at all, 5= always)- Given at baseline and monthly

1. Feeling compassion for others exhausts your mental energy, which you need to refuel afterwards.

2. After feeling sincere compassion for others, your emotional energy is depleted.

3. Feeling compassionate is emotionally energizing, and you are able to immediately start feeling compassionate toward other people, too.

4. Even after feeling deeply compassionate, you can continue to feel compassionate towards others.

5. After feeling deeply compassionate for someone, you have to recover your emotional energy to feel compassionate again.

6. After sympathizing for someone deeply, you are further motivated to feel compassion for other people.

7. Feeling deeply compassionate for someone makes it difficult to feel compassionate for others who deserve compassion.

8. When situations accumulate that make you feel compassionate, it gets more and more difficult to feel true compassion.

9. If you have just felt compassionate, you feel strengthened and you can continue to feel compassionate.

10. It is particularly difficult to feel compassionate for a new person after just feeling compassionate right before for someone else.

11. Feeling compassionate makes you become even better able to feel compassionate toward new issues.

12. Your capacity to feel compassionate is not limited.

STAI-6 (Given monthly Only) (1= not at all, 2= somewhat, 3= moderately, 4= very much)

Read each statement and then click the most appropriate number to indicate how you feel right now, at this moment. There are no wrong answers. Do not spend too much time on any one statement but give the answer which seems to describe your present feeling the best.

1) I feel calm

2) I am tense

3) I feel upset

4) I am relaxed

5) I feel content

6) I am worried

STAI- Full (Only given at baseline)

COVID and public health perceptions (baseline and monthly)

COVID-19 Threat (1 = “Strongly disagree” to 7 = “Strongly agree” scale)

How much of a threat, if any, is the coronavirus outbreak for.....

• The rights and freedoms of the U.S. population as a whole

• What it means to be American

• American values and traditions

• American democracy

• The maintenance of law and order in America

• Your personal health

• The health of the U.S. population as a whole

• Your personal financial safety

• The U.S. economy

• Day-to-day life in your local community

Support for Public Health Initiatives (1 = “Strongly disagree” to 7 = “Strongly agree” scale)

• The social distancing restrictions being put into place to stop the spread of Covid-19 are doing more harm than good.

• We need to prioritize going back to our normal routines as soon as possible, regardless of COVID-19’s spread.

• Right now the most important thing we can do is take all measures possible to stop the spread of COVID-19.

• It is essential that we strictly practice social distancing as a nation, until health care experts say otherwise.

How often did you wear a mask or other face covering in the past 2 weeks when you were doing each of the following activities

Scale: (N/A, as i never did this activity; never; some of the time; most of the time; every time)

- Getting fresh air or exercising outdoors
- Shopping for groceries or other necessities
- Meeting people social outside
- Meeting people social indoors
- Doing work or school work outside my home

In the last 2 weeks, how many times have you done each of the following activities?

Scale: (N/A, as i never did this activity; never; some of the time; most of the time; every time)

- Gone out to a restaurant, bar, or other indoor place where people gather
- Gone inside a friend, neighbor, or relative’s residence that is not your own
- Had visitors such as friends, neighbors or relatives inside your residence
- Remained in your residence at all times, except for essential activities or exercise
- Had close contact – within about 6 feet – with people who do not live with you
- Shopped inside stores for items that I likely could have ordered online or bought using a pickup option

Student Related Questions (11 items)- Given at baseline and monthly

Social fit. We assessed participants’ social fit in college using a six item scale (Walton & Cohen, 2007). On a scale from 1(strongly disagree) to 7(strongly agree), participants rated their agreement with the following items:

(1) I feel like I belong as a student at the University of Michigan

(2) I feel like I fit in with the academic community at University of Michigan

(3) If my parents were to visit me or when they have visited me at University of Michigan, I would feel or have felt comfortable introducing them to my friends

(4) I feel a part of the University of Michigan community

(5) I expect that the social experience at University of Michigan will be difficult for me

(6) In the future, I could see myself having a lot of friends at University of Michigan

Academic identification. To assess academic identification, two items were used (Walton & Cohen, 2011). Specifically, on a scale from 1(not at all important) to 7(essential to who I am), students answered the following questions:

(1) How important is academic success to you?

(2) How important is being a college student to you?

Tendency to seek college resources. To measure participants’ tendency to seek out college resources, we asked the following three questions: Sliding scale from 0 to 5 times.

(1) Since you started at University of Michigan in a typical month, how many times have you emailed a professor to ask a question?

(2) Since you started at the University of Michigan, how many times have you met with a professor outside of class?

(3) Please estimate how many times you have gone to the writing center since you started at the University of Michigan.

Monthly Questionnaires

Questions related to the experience and value from participating in the study; questions related to beliefs and expectations.

1. Have you moved in the last month?

a. Yes, moved out of Ann Arbor

b. Yes, moved to Ann Arbor

c. Yes, not Ann Arbor related

d. No, I have not moved

2. Have you traveled in the last month?

a. Yes, within the state that I live

b. Yes, outside of the state, but within the country

c. Yes, outside the country

d. No, I have not traveled

1. What is your current housing?

a. Living at home with parents or family members

b. Living on/near campus in a house

c. Living on/near campus in an apartment

d. Living on campus in a dorm

e. Living further than 1 hour drive away from Ann Arbor, but not at family’s home

f. Other; please specify _______________

4. What is the makeup of the people living in your household (check all that apply and include number)

a. Roommates (Same room):___

b. Roommates/housemates (different room): ___

c. Family: ___

d. I live alone

5. I feel stressed about the COVID-19 pandemic.

a. Strongly agree (1)

b. Somewhat agree (2)

c. Neither agree nor disagree (3)

d. Somewhat disagree (4)

e. Strongly disagree (5)

6. I feel optimistic about the future

a. Strongly agree (1)

b. Somewhat agree (2)

c. Neither agree nor disagree (3)

d. Somewhat disagree (4)

e. Strongly disagree (5)

7. I feel I am likely to become infected with the novel coronavirus.

a. Strongly agree (1)

b. Somewhat agree (2)

c. Neither agree nor disagree (3)

d. Somewhat disagree (4)

e. Strongly disagree (5)

f. I have already had a positive COVID-19 test

8. I feel safe from COVID-19.

a. Strongly agree (1)

b. Somewhat agree (2)

c. Neither agree nor disagree (3)

d. Somewhat disagree (4)

e. Strongly disagree (5)

9. I believe I will remain healthy with respect to COVID-19

a. Strongly agree (1)

b. Somewhat agree (2)

c. Neither agree nor disagree (3)

d. Somewhat disagree (4)

e. Strongly disagree (5)

Social Rhythms Baseline Survey

1. What is your age, in years?

2. Please select your gender?

3. If they select "Prefer to self describe" then they also get a question: "Please enter your gender" with a text box.

4. What is your race?

5. Did you social distance for COVID-19, or are you currently doing so?

6. When did you begin social distancing for COVID-19?

7. Have you ended Social distancing for COVID-19

8. When did you end social distancing for COVID-19?9. What type of Urban or Rural environment do you live

General Anxiety Disorder-7 and Patient Health Questionnaire- 9

PHQ-9:

How often have you been bothered by the following over the past 2 weeks?

1. Little interest or pleasure in doing things?

a. Not at all

b. Several days

c. More than half the days

d. Nearly every day

2. Feeling down, depressed, or hopeless?

a. Not at all

b. Several days

c. More than half the days

d. Nearly every day

3. Trouble falling or staying asleep, or sleeping too much?

a. Not at all

b. Several days

c. More than half the days

d. Nearly every day

4. Feeling tired or having little energy?

a. Not at all

b. Several days

c. More than half the days

d. Nearly every day

5. Poor appetite or overeating?

a. Not at all

b. Several days

c. More than half the days

d. Nearly every day

6. Feeling bad about yourself- or that you are a failure or have let yourself or your family down?

a. Not at all

b. Several days

c. More than half the days

d. Nearly every day

7. Trouble concentrating on things, such as reading the newspaper or watching television?

a. Not at all

b. Several days

c. More than half the days

d. Nearly every day

8. Moving or speaking so slowly that other people could have noticed? Or so fidgety or restless that you have been moving a lot more than usual?

a. Not at all

b. Several days

c. More than half the days

d. Nearly every day

9. Thoughts that you would be better off dead, or thoughts of hurting yourself in some way?

a. Not at all

b. Several days

c. More than half the days

d. Nearly every day

GAD-7:

How often have you been bothered by the following over the past 2 weeks?

1. Feeling nervous, anxious, or on edge?

a. Not at all

b. Several days

c. More than half the days

d. Nearly every day

2. Not being able to stop or control worrying?

a. Not at all

b. Several days

c. More than half the days

d. Nearly every day

3. Worrying too much about different things?

a. Not at all

b. Several days

c. More than half the days

d. Nearly every day

4. Trouble relaxing?

a. Not at all

b. Several days

c. More than half the days

d. Nearly every day

5. Being so restless that it’s hard to sit still?

a. Not at all

b. Several days

c. More than half the days

d. Nearly every day

6. Becoming easily annoyed or irritable?

a. Not at all

b. Several days

c. More than half the days

d. Nearly every day

7. Feeling afraid as if something awful might happen?

a) Not at all

b) Several days More than half the daysNearly every day

Exit survey

Part 1: Indepth- COVID Screening

Have you ever been tested, or are currently scheduled to be tested for COVID? (Including serology)

a. Yes

b. No

If yes: How many times have you been tested for COVID?

a. Once

b. Twice

c. Three times

d. Four times

e. Five times

f. Greater than five times

g. I have not been tested for COVID

If no: Did you have any of the following symptoms but never received testing for COVID?

a. Fever

b. Chills

c. Shortness of breath

d. New or worsening cough

e. Sore throat

f. Body aches

g. Vomiting

h. Diarrhea

i. Loss of smell

j. Loss of taste

k. Other; please specify

l. None of the above

What date did your symptoms begin?

The following questions will be asked about each test the participant has selected that they have received (Up to 5 tests)

What prompted you to be tested for COVID?

a. Symptoms

b. Contact with someone who tested positive

c. Contact with someone who showed symptoms

d. Asymptomatic screening program

e. Other; please specify

What type of testing was done?

a. PCR and/or rapid test

b. Antibody test

c. Other; please specify

What was the approximate date of your test?

What were the results of your test?

a. Positive

b. Negative

c. Pending

Did you have any of the following symptoms that prompted you to be tested for COVID? Please select all that apply.

a. Fever

b. Chills

c. Shortness of breath

d. New or worsening cough

e. Sore throat

f. Body aches

g. Vomiting

h. Diarrhea

i. Loss of smell

j. Loss of taste

k. Other; please specify

l. None of the above

What date did your symptoms begin?

Part 2: COVID Compassion and Fatigue

Scale 1 (all strongly disagree to strongly agree 1 to 7):

1. Over the course of the COVID-19 pandemic, my compassion response has weakened over time
2. It has become harder to truly care about the COVID-19 pandemic as more and more people have become affected.
3. I feel emotionally exhausted as a result of the COVID-19 pandemic.
4. During the COVID-19 pandemic, I have felt more and more compassion as I am exposed to more people suffering.
5. Over time, my motivation to address people’s suffering from COVID-19 has increased

#### Scale 2 (rarely/never=1 to very often=10)

To what extent have you experienced the below thoughts during the COVID-19 pandemic

1. Flashbacks connected to other people’s suffering
2. Troubling dreams about other people’s suffering
3. Intrusive thoughts about the world's problems
4. Suddenly recalled an experience of another person's suffering
5. Losing sleep over other people’s suffering
6. Feeling trapped due to helping other people with their suffering
7. Sense of hopelessness about the world's problems
8. Feeling tired due to considering other people's suffering
9. Feeling depressed due to considering other people's suffering
10. Sense of worthlessness as an individual
11. Feeling like a "failure" at helping other people
12. Thoughts about not achieving my goals
13. Unsuccessful at separating my own well-being from the wellbeing of others

Scale 3 (two measures about predictors of compassion fatigue):

1. To what extent have you provided care or support for people suffering from COVID-19’s negative effects (e.g., illness, loss of a loved one, job loss)?
   1. Scale: I have not done this at all, I have done this on rare occasions, I have done this some, but not frequently, I have done this a lot, but not always, I do this nearly every day or more
2. How frequently do you engage with news media that talks about the suffering and hardship that COVID-19 has caused?
   1. Scale: more than once a day, once a day, several times per week, once a week, once every two weeks, once a month, less than once a month
3. COVID-19 has made it difficult for me to care as much about other problems in the world that are unconnected to COVID-19

Scale: strongly disagree to strongly agree, 1-7 points

COVID Vaccination

When the COVID-19 vaccine is available to you, do you intend to get the vaccine?

a. I definitely will not get the vaccine

b. It's possible I'll get the vaccine, but I probably will choose not to.

c. I probably will get the vaccine, but it's possible that I will choose not to.

d. I definitely will get the vaccine or have already gotten the vaccine

If you have already received the first dose of the COVID-19 vaccine or have an appointment to receive your COVID-19 vaccine, please indicate the date of your first dose. ____________

If you have already received the second dose of the COVID-19 vaccine or have an appointment to receive your second dose of the COVID-19 vaccine, please indicate the date of your second dose __________

If you have already received your COVID vaccine which did you receive?

a. Pfizer

b. Moderna

c. Johnson & Johnson

d. Astra Zeneca

e. Other; please specify
